# Attitudes of Healthcare Workers towards Older People in a Rural Population: A Survey Using the Kogan Scale

**DOI:** 10.1155/2011/352627

**Published:** 2011-04-14

**Authors:** Mandy Doherty, Elizabeth A. Mitchell, Siobhan O'Neill

**Affiliations:** ^1^Carndonagh Community Hospital, Donegal, Ireland; ^2^School of Nursing, University of Ulster, Londonderry BT487JL, UK; ^3^Psychology Research Institute, University of Ulster, Northland Road, Londonderry BT487JL, UK

## Abstract

With the global trend towards an increasingly ageing population, it is clear that nurses need to be equipped with the knowledge and skills to fulfil significant roles in responding to future health and support needs. This paper reports the results of a survey that aimed to identify and evaluate the attitudes of nurses, healthcare assistants, and nursing students towards older people. The survey was undertaken in a rural county in the Republic of Ireland. It is reassuring that in our study, we found that these healthcare workers hold positive attitudes towards older people. In addition, we found that study to a higher level at university appears to mitigate towards holding more positive attitudes, and this is an important finding in light of the shift towards nursing as an all-graduate profession.

## 1. Introduction

Healthcare workers are considered to be at particular risk of developing ageist attitudes because they are exposed to a disproportionate percentage of ill or dependent older people. Research demonstrates that many factors have influence on healthcare workers' attitudes towards older people, including age [1], gender [1–3], education [4–6], exposure to well older people [7–10], area of practice [11–14], and professional socialization [15]. An attitude is an evaluation of something or someone on a continuum of like to dislike or favourable to unfavourable [16]. Ageism has been defined as “a systematic stereotyping of discrimination against people because they are old” [17]. Ageism is perpetuated by the portrayal of older people as frail, ill, suffering mental deterioration, poor and dependent, and the alternative portrayal of living affluent life styles and scrounging off the welfare state [18]. In addition, budget constraints in the UK and Ireland which currently pitch health services against social services over costs for supportive care packages negate against timely discharge from hospital and reinforce an overreliance on family careers for support. These consequences are likely to further reinforce the portrayal of older people as a burden on society. Stereotypes such as these are in contrast with the reality that the majority of older people lead fit and independent lives [19]. Older people consider that inadequate housing, low income, enforced retirement, and deficiencies in nursing home care are evidence of ageism [20]. In addition, the National Council of Ageing and Older People study undertaken in 2005 in the Republic of Ireland concluded that indirect discrimination, such as barriers to access of services, inadequate transportation, excessive waiting in accident and emergency and outpatient departments, and underfunded community services, is widespread and frequent [21]. 

Internationally, older people care services have difficulty in attracting and retaining staff [22, 23]. The status afforded by health care workers to older people in their care has received attention from researchers [20, 24, 25]. Minichiello et al. provided evidence of older people being neglected or treated as unimportant [20], and more recently Mitchell and McCance indicated that older people perceive that they are commonly denied an active role in making decisions about their health and lives [25]. Reports of elder abuse cases in care homes in the Republic of Ireland [26], mortality rates and neglect of older patients in a UK NHS hospital [27], and substandard care of patients with dementia [28] have indicated inadequate training, poor communication and management, and insufficient access to specialist services and rehabilitation therapy as contributing factors. The O Neill report of systematic abuse of residents in the Leas Cross nursing home in the Republic of Ireland highlighted that managers and clinical leaders did not recognise the care required to meet the needs of their residents [26]. In addition, O Neill identified failures at Area Health Board, the Health Service Executive (HSE), and government levels for the quality of care the residents received. It was contended that the low priority afforded by these public organisations to the needs of older people and their failure to provide adequate policy and legislation had direct consequences for care standards. The quality of care that older people receive in health services reflects the innate nature of ageism within society. Indeed, chronic underfunding and low priority given to the development of services for older people is considered to profoundly influence how healthcare workers think about working with older people [12, 26]. 

The Republic of Ireland has a population of 4,203,200, and the proportion of its population aged 65 years and above is 11.0% [29]. Initiatives in 2005 to counter ageism and elder abuse in Ireland included a rise in the state old age pension to reduce the poverty risk in its older population from 27% to 20% and an annual “say no to ageism” week [30]. The Health Service Executive in 2008 introduced mandatory training on “Recognising and Responding to Elder Abuse in Residential Care Setting,” which focused on discrimination in general, but particularly ageism [31]. The “Fair Deal” legislation introduced in 2009 espouses principles of equality and fairness in access to services but acknowledges that independent financial resources continue to have significant influence on personal choice and timely supportive services [32]. Ageing demographics is a global phenomenon, and the Republic of Ireland is anticipating a 25% increase in its population aged 65 years and over, from year 2004 to 2026 [29]. Given the rising percentage of older people using hospital and community services, and almost weekly accounts in the media of substandard care of older people, it is important that the attitudes of healthcare workers towards older people are re-examined.

## 2. Material and Methods

### 2.1. The Study

Our study was undertaken in a rural county, where common to other rural counties of Ireland work outside the home, and the migration of younger family members to urban areas to seek work is challenging care services for older people. In this county, health care is managed through one acute regional hospital, nine community hospitals, two community nursing units, and approximately 58 public health nurses. Older people in receipt of nursing care will encounter ward managers, registered staff nurses, healthcare assistants, public health nurses, community-registered nurses, and student nurses, and the decision was taken to include all these groups in a survey of attitudes towards older people. The aim of this study was to explore the attitudes held by these health care workers towards older people in this rural population. 

### 2.2. Objectives

- To measure, using Kogan's Attitudes Towards Older People Scale [KOP], the attitudes of health care workers towards older people.
- To compare scores on KOP across groups of health care workers categorised by role title, length of service in current role, and across work place setting.
- To explore the relationship between highest education attainment and KOP scores.
- To make recommendations to inform education, research, and practice.

The design of the study was a survey of attitudes of healthcare workers towards older people. Attitudes towards older people were measured using the Kogan's Attitudes towards Older People Scale [KOP], developed by Kogan in 1961 [33]. In developing the scale, Kogan assigned older people the status of a minority group, and the items in the scale originated within ethnic minority stereotype research. The KOP is a 34-item Likert type scale with 17 matched positive and negative statements; an example of negative-positive item pair is “most older people tend to let their homes become shabby and unattractive” and “most old people can generally be counted on to maintain a clean, attractive home.” It must be acknowledged that the Kogan Scale was developed in the early 1960s, and it has been subject to criticism. McLafferty has suggested that the scale measures societal attitudes but does not take into account the particular context in which nurses meet older people [34], and Iwasaki and Jones highlight the diverse nature of older life and that neither positive nor negative images can reveal the ambiguous nature of people's attitudes, views, and experiences of ageing [35]. However, the scale has been used extensively to measure attitudes towards older people across a range of professional groups [3, 6, 8, 10, 11]. Kogan investigated the scale's reliability and reported Spearman-Brown reliability coefficients ranging from 0.66, to 0.83, and interscale item correlations ranging from 0.46 to 0.52 [33]. In addition, Kogan contended concurrent validity by the use of measures of antiauthoritarian and antiminority attitudes. The reliability of KOP has since been confirmed by other researchers [3, 6, 8, 10, 11]. The KOP in our study was scored on a 6-point Likert scale: 6: highly positive, 5: positive, 4: slightly positive, 3: slightly negative, 2: negative, and 1: highly negative. The negative statements were reverse scored, so that higher scores were attributed to more positive attitudes. The minimum score possible was 34, and the maximum score possible was 204. 

The questionnaire was prepared encompassing two sections.  Section 1 was designed to capture biographical and contextual data such as gender, job title, length of time in current role, work setting and highest education qualification, and completion of a “healthcare/carers” course and “care of older people” course.  Section 2 comprised the “Kogan Attitudes towards Older People Scale” [33]. A participant information letter was provided, and the voluntary nature of completion was stated. Consenting participants completed the questionnaire, sealed it in the envelope provided, and placed it in a marked container in the practice setting office for collection. The number of staff on duty during the designated 24–hour data collection period in year 2009 was ascertained from service managers and duty rosters to establish the questionnaire response rate.

## 3. Sampling and Ethical Considerations

The participants were employed within Health Service Executive sectors as ward managers, registered nurses, public health nurses, healthcare assistants, and student nurses. The participants will be referred to collectively as healthcare workers. The sampling strategy adopted was a convenience sample of health care workers in the work settings of an acute regional hospital, community hospitals, nursing units, and in the community. In the regional hospital, the sample comprised of all these healthcare workers from the three general medical wards and two surgical wards and its one rehabilitation ward. In the community, the sample was comprised of all healthcare workers rostered to work in the county's nine community hospitals and its two nursing units during the 24-hour data collection period. Directors of nursing and managers agreed to distribute the questionnaires during the collection period in 2009. There are approximately 58 public health nurses based in health centres throughout the county. Owing to the large geographical nature of the county, a random sample of twenty public health nurses were sent the questionnaire by post, with a stamped addressed envelope for response.

The researcher received approval to proceed with the study from the University of  Ulster's Research Ethics Committee and the Ethics Committee in the regional hospital. The regional hospital has responsibility for both hospital and community nursing services in the county. The main ethical implications of this research were informed consent, confidentiality, and anonymity of participants. The service managers for the acute hospital, the community nursing services, the community hospitals, and nursing units were contacted and requested in writing for permission to distribute questionnaires to their health care workers, which was granted. There was a minimal amount of risk associated with this research, as the participants were staff employed by the Health Service Executive. It was anticipated that the research would help to identify those categories of healthcare workers who may express ageist attitudes. Whilst there is a link between attitude and predicted behaviour, expressing ageist attitudes does not mean that a group of staff do not give high-quality care. If ageist attitudes were expressed, then recommendations for education interventions for these staff would be proposed. 

## 4. Data Analysis

The questionnaires were coded jointly by the first and second author to ensure consistency of analysis, and standard schemes were used to compute the scores for KOP. Data was analysed using SPSS for Windows v.17 (SPSS Inc., Chicago, IL). The KOP was computed by using a 6-point Likert approach, in which the more positive attitudes towards older people achieved a higher score. The KOP was totalled, and cross-referenced to the returned questionnaires to address any errors or missed data coding. Missing data on SPSS was replaced with the midpoint number 3.5, a strategy supported by Palliant [36]. Descriptive statistics were used to explore the characteristics of the sample. For statistical tests, the level of significance considered appropriate for this study was a value for *P* of  ≤.05. *t*-tests were conducted to compare mean attitude scores across two groups. One-way analysis of variance with post hoc comparisons were conducted to examine whether attitudes were significantly different between groups of health care workers.

## 5. Results

A total of 303 questionnaires were distributed to health care workers, and 190 returned the completed questionnaires. The study had an overall response rate of 62.2%, which is comparable to response rates in similar studies of 57% [6] and 69% [11]. Three questionnaires with five or more missing item data out of a total of 34 were withdrawn from the study. Five returned questionnaires had some missing data in Section 1, which requested information on occupation and qualifications; these were not excluded from the KOP analysis and the missing data remained. The prevalence of health care worker occupations were registered staff nurses 85 (45.5%), health care assistants 49 (26.2%), ward managers 21 (11.2%), student nurses 19 (10.2%), and public health nurses 11 (5.9%). The prevalence rate of highest educational attainment was ascertained as outlined in Table 1, and it was observed that 33.7% of nurses were university graduates having attained a nursing degree, higher diploma, or MSc. In addition, 83% of the healthcare assistants in this survey had either undertaken a “healthcare/carers” course, a “care of older people” course, or both. 

The distribution of attitude scores was subjected to tests of normality. Kolmogorov-Smirnov statistic equal to 0.200 was a nonsignificant result, and visual inspection of the distribution of scores indicated normality (Table 2). The internal consistency of the KOP was tested by using Cronbach alpha. The measure was found to be reliable on the basis of the data obtained in the questionnaires (Cronback *a* = 0.751). The variation of intensity of attitudes was illustrated by dividing the potential range of scores into six equal categories, and this showed that health care workers held positive attitudes towards older people; indeed 97.3% of scores fell into the “slightly positive” to “very positive” score ranges (Table 3).

**Figure 1:**
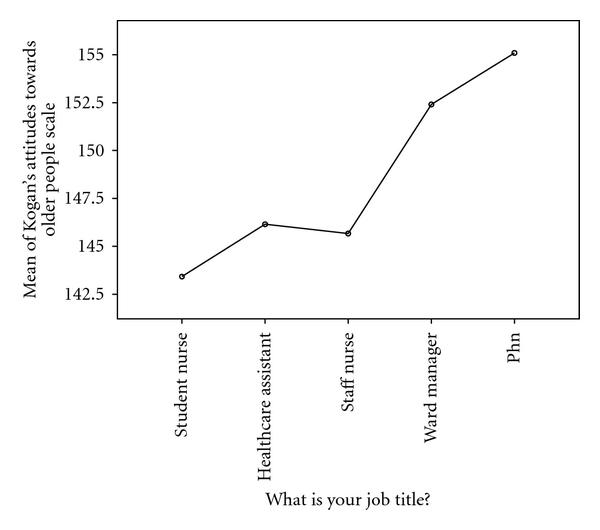
Kogan's attitudes towards old people scale scores by job title.

An independent sample *t*-test was used to compare KOP mean attitude scores between males (*N* = 19) and females (*N* = 167). Difference across gender was not significant (*P* = .198). Participants were then categorised in 5 groups according to their job titles: ward manager, registered nurse, public health nurse, student nurse, and healthcare assistant. A one-way between-groups analysis of variance was conducted to explore differences in attitudes between these groups. Levene statistic was 0.202, indicating that we had not violated the assumption of homogeneity of variance. Although ward managers and public health nurses held more positive attitudes (Figure 1), post-hoc comparisons performed using the Tukey HSD test failed to detect a significant difference in KOP scores between the groups (*P* = .135).

A one-way between-groups analysis of variance was conducted to explore the attitudes of participants categorised into 7 groups by length of service in current role: those who had worked ≤5 years; 6–10 years; 11–15 years; 16–20 years; 21–25 years; 26–30 years; ≥31 years. Levene statistic was 0.953, indicating that we had not violated the assumption of homogeneity of variance. KOP scores between these groups were not significantly different [*F*(6,179) = 1.8, *P* = .106]. An independent sample *t*-test was conducted to explore attitudes between health care workers who worked in acute care settings and those who worked in the community/community hospital settings and mean attitudes were not significantly different between these two groups (*P* = .248). An independent sample *T*-test was conducted to compare KOP scores for those who had attained a university degree, higher diploma, or MSc and those who had not attained qualifications at university. There was a significant difference in scores for university graduates (*M* = 149.34, SD = 15.4), compared to those who had not attained university qualifications [*M* = 144.88, SD = 12.45; *t*(161) = 2.02, *P* = .044].

## 6. Discussion

This study set out to explore the attitudes held by groups of health care workers towards older people in a rural population. It was reassuring to find that health care workers in this rural county in the Republic of Ireland generally held positive attitudes towards older people. Our study did not detect significant differences in attitude scores measured by KOP across gender, job title, length of service in current role, and work place setting. In contrast, our study did detect a significant difference in scores for university graduates, when compared to those who had not attained a university qualification, with university study associated with more positive attitudes.

Research indicates that health care workers may hold negative attitudes towards the structural context of work and the restrictive practices that can pervade in older people care settings. Research which supports this context dimension [12, 13] suggests that attitudes can be negatively influenced by the underresourced care environments experienced when working with older people. Our study did not detect a significant difference in KOP scores between health care workers who worked in acute care services and those in community, community hospital settings, or nursing units. Hope [11] and later McLafferty and Morrison [14] reported that health care workers in care settings for older people had more positive attitudes towards older people than those working in more acute care settings. However, more recently, and similar to our findings, Gallagher et al.'s research [6] did not detect a significant difference in attitudes across work setting. Organisational change, health service reform, early supported discharge and ageing demographics in the last two decades, and mediating towards a higher prevalence of older people in acute care wards mean that in almost all care settings, health care workers are increasingly working with older people, and perhaps this explains the similar favourable attitudes. 

Over 83% of the healthcare assistants in our study had either undertaken a “healthcare/carers” course, a “care of older people” course, or both. Healthcare assistants provide much of the face to face care with older people, but their attitudes towards older people have received only scanty attention from researchers [4, 6]. In our study, no significant KOP score differences were found between staff nurses and healthcare assistants, which contrasts with previous research that reported that healthcare assistants held more negative attitudes towards older people than registered nurses [6]. Perhaps the recent Irish government initiatives to combat ageism and the high uptake of “healthcare/carers” courses and “care of older people” courses by healthcare assistants have led towards the more positive attitudes. 

It is perhaps significant that 33.7% of participants were university graduates having obtained a degree, higher diploma, or a master's degree. Health care workers are a stable workforce in this rural county, and full-time job vacancies can be rare. Individual staff may consider that university academic attainment affords them greater opportunities for career progression than others without university qualifications. Education has been identified in previous studies as a predictor for attitudes towards older people [5, 37]. Our finding that staff who attained a university qualification had significantly higher mean KOP scores than those health care workers who had not attained university qualifications is particularly interesting in light of the shift towards nursing becoming an all-graduate profession. 

The research evidence relating to education and attitudes towards older people is complex, and perhaps this is not surprising, when educational attainment is not a guarantee that individuals have received sufficient education and training in the needs of and care required by those who are older. Nurses who work with older people are critical of both staff in acute care sectors and educators of nursing students, for not fully addressing the needs of older people in education programmes [13, 14]. In addition, there has been criticism that nurse educators fail to counter negative perceptions of working with older people as a future career option of nursing students [15]. Indeed, Stevens and Crouch considered that nurse educators perpetuated the view that acute care was more demanding of skill and knowledge [15]. Criticisms of nurse education has been reinforced by the recent Alzheimer's Society survey of 1100 nurses in the UK which reported that 80% of participants felt they had been inadequately trained to work with people with dementia [28]. 

Our findings suggest that public health nurses hold more positive attitudes towards older people than registered nurses, nursing assistants, and students, although it must be noted that differences across role title were not significant. In the Republic of Ireland, public health nurses commonly hold three professional qualifications: general nursing, midwifery, and public health nursing. Public health nurses are community based, and their work is with all levels of that community, working with individuals, families, and groups. They are autonomous practitioners who prioritise their workloads, in their roles as managers, clinicians, and health promoters [38]. It is conceivable that in this rural county, the public health nurses know the older person they are working with, not only as an older person but possibly as a neighbour, distant relative, or friend. In addition, as they are embedded in the community, the person being supported is real and likely to be perceived as living independently. This community embedded “knowing the person” might be very different from the experience of some of the healthcare workers who primarily work in hospitals, which may partly explain why public health workers hold the most positive attitudes. However, ward managers working in both acute hospital settings and the community nursing units also generally held more positive attitudes than registered nurses and health care assistants. It is likely that as 61.9% of ward managers had studied to degree, higher diploma, or masters, that higher education attainment is also a factor that links ward managers and public health nurses in terms of more positive attitudes. 

There are a number of limitations in this study that would need to be addressed if this study was to be undertaken again. The study did not ask respondents their age. As age is one of the significant influencing factors on attitudes [1], it would have been pertinent to see if that finding was replicated in this study. However, the attitude scores of participants categorised by length of service in current role were scrutinised, and significant differences were not detected. A six-point Likert scale was chosen, similar to Lookinland and Anson [3] and Haight et al. [7]. However the authors of this study suggest that missing data could be managed more simply, by using a whole midpoint number, such as in a 5-point Likert scale used by Ryan et al. [10]. These points indicate the importance of undertaking a pilot study with all questionnaire tools, which would have given the researcher the opportunity to make informed amendments.

## 7. Conclusions

Our study has shown that the vast majority of the participants which included nurses, nursing assistants, and students hold positive attitudes, towards older people. In addition, we found that study to a higher level at university appears to mitigate towards holding more positive attitudes and this is an important finding in light of the shift towards nursing as an all-graduate profession. As previously stated, the study was undertaken in a rural county in the Republic of Ireland, with a generally stable nursing workforce, and it is possible that healthcare workers are regularly caring for older people they have come to know as individuals. With the global trend towards an increasingly ageing population, it is clear that nurses need to be equipped with the knowledge and skills to fulfil significant roles in responding to future health and support needs. The recent initiatives in the Republic of Ireland to counter ageism and elder abuse [30–32] are commendable, and in addition the high update of “carers” and “care of older people” courses by nursing assistants may be promoting more positive attitudes. Budget constraints in the UK and Ireland currently pitch health services against social services and stall the development of supportive care packages, with resultant delays in discharge from acute hospital care and overreliance on family carers. These consequences are likely in the future to further reinforce the portrayal of older people as a burden on society. The current reports of elder abuse, neglect, and substandard care receiving media coverage are also in danger of undermining those healthcare workers who are attempting to provide quality care in challenging times. Despite this context, it is reassuring that in our study, nurses and nursing assistants hold positive attitudes towards older people.

## Figures and Tables

**Table 1:** Highest education qualification.

Valid	Frequency	Percent
No education qualification	3	1.6%
Junior cert/O level	7	3.7%
Leaving cert/A level	34	18.2%
Registered nurse training	56	29.9%
Degree/Higher Diploma/MSc	63	33.7%
Missing	24	12.8%

Total	187	100%

**Table 2:** Test of normality.

	Kolmogorov-Smirnov		
Tests of normality	Statistic	d.f.	Sig.
Kogan's Attitudes towards Older People Scale [KOP]	0.048	187	0.200*

*This is a lower bound of the true significance.

**Table 3:** Kogan's attitudes towards older people scale scores.

	Range of scores	Score *n* (%)
Very negative	34–62.3	0 (0)
Negative	62.4–90.7	0 (0)
Slightly negative	90.8–119.1	5 (2.7)
Slightly positive	119.2–147.5	95 (50.8)
Positive	147.6–175.9	83 (44.4)
Very positive	176–204	4 (2.1)
